# Variation of outdoor illumination as a function of solar elevation and light pollution

**DOI:** 10.1038/srep26756

**Published:** 2016-06-07

**Authors:** Manuel Spitschan, Geoffrey K. Aguirre, David H. Brainard, Alison M. Sweeney

**Affiliations:** 1 Department of Psychology, University of Pennsylvania, Philadelphia, Pennsylvania 19104, USA; 2 Department of Neurology, University of Pennsylvania, Philadelphia, Pennsylvania 19104, USA; 3 Department of Physics and Astronomy, University of Pennsylvania, Philadelphia, Pennsylvania 19104, USA

## Abstract

The illumination of the environment undergoes both intensity and spectral changes during the 24 h cycle of a day. Daylight spectral power distributions are well described by low-dimensional models such as the CIE (Commission Internationale de l’Éclairage) daylight model, but the performance of this model in non-daylight regimes is not characterised. We measured downwelling spectral irradiance across multiple days in two locations in North America: One rural location (Cherry Springs State Park, PA) with minimal anthropogenic light sources, and one city location (Philadelphia, PA). We characterise the spectral, intensity and colour changes and extend the existing CIE model for daylight to capture twilight components and the spectrum of the night sky.

During the 24-hour cycle, ambient illumination changes as a function of the Earth’s rotation in both *intensity* and *spectral composition*. During the day and before twilight, the ambient illumination is between 1,000,000 (10^6^) and 100,000,000 (10^8^) times brighter than at night^1^. Starlight on a clear night has a brightness of ~0.001 lux, and moonlight ~0.2 lux^1^. In comparison, sunlight may be as intense as 100,000 lux^1^. The ambient light level also depends on the presence of clouds and haze, and may vary minute-to-minute due to cloud cover and atmospheric turbidity.

In the 1960s, coordinated efforts were made to describe the spectral power distribution of daylight. In their seminal work, Judd, *et al.*^2^ subjected a set of 622 measured daylight spectral power distributions to a dimensionality reduction technique and derived three basis functions (termed S0, S1 and S2 in the original work and below) which account for much of the variance in the dataset. These were later accepted as the Commission Internationale de l’Éclairage (CIE) daylight model (henceforth called the ‘CIE daylight model’), and are widely used for modelling and synthesizing the spectral power distributions of daylight^3^.

Ambient illumination intensity changes systematically and most rapidly around twilight, decreasing (at dusk) or increasing (at dawn) as a function of decreasing or increasing solar elevation (*θ*_*s*_)^1^,^4^. When the Sun has set below the horizon at twilight (*θ*_*s*_ < 0°) and no longer directly illuminates the Earth, the light in the sky results in part from refraction and scattering of the Sun’s rays in the upper atmosphere. Twilight is classified into three distinct phases according to solar elevation and the prevailing visibility conditions due to the illumination level: a) *civil twilight* (−6° < *θ*_*s*_ < 0°), when terrestrial objects can still be distinguished by human observers, b) *nautical twilight* (−12° < *θ*_*s*_ < −6°), when only object outlines are visible, and c) *astronomical twilight* (−18° < *θ*_*s*_ < −12°), when the illumination level is low enough such that stars and other astronomical objects are available for observation^5^.

During twilight not only does the intensity of the illumination change, but so does the spectral composition (colour), giving rise to vivid phenomena visible to the human eye at and before twilight^6^,^7^,^8^, such as the yellowish twilight arches during civil twilight and the purple and red sky during nautical twilight. With ever decreasing solar elevations, the short-wavelength content of ambient light becomes enriched due to the increasing amount of ozone absorption caused by the increased path length of solar light through the atmosphere and so-called Chappuis filtering of green/yellow light^9^. This period is informally termed the “blue hour” in photography. The accuracy with which the 3-component CIE daylight model captures variability in spectral composition at twilight, or more generally as a function of solar elevation, is not known. There is evidence that twilight exhibits statistical regularity^1^,^10^,^11^, but no efforts have been made to extend the CIE daylight model to capture twilight illumination.

The night sky has changed in recent human history. Since the invention of electric light in the 19^th^ century, anthropogenic light sources illuminating developed areas have become more and more prevalent, effectively extending the availability of light well beyond twilight. Bright night skies due to urban and suburban outdoor illumination have risen in the second half of the 20^th^ century^12^; this is referred to as *light pollution*. Much light pollution comes not from consumer-grade incandescent and fluorescent lights but rather from mercury bulbs and high- and low-pressure sodium lamps commonly used for outdoor lighting^1^. Artificial lightning not only changes the amount of ambient light but also its spectral composition. How anthropogenic light affects the spectral and intensity changes of ambient illumination as a function of the solar day has not been examined. Measurements of this may inform studies of potential adverse effects of light pollution on ecology and health^13^,^14^.

In this study, we investigate the feasibility of extending the CIE daylight model to capture non-daylight outdoor illumination. We characterised the changes in intensity and spectral composition of downwelling illumination across daylight, twilight, and night. We collected illumination spectra under varying lunar and solar configurations in July 2014 at two measurement sites in the Northern hemisphere: one site with minimal light pollution (Cherry Springs State Park, Northern Pennsylvania, USA; henceforth called *Rural*), and one in an urban setting (Philadelphia, PA, USA; henceforth called *City*). We use the data to ask how well the CIE daylight model captures illumination during twilight and at night at both locations, and show that deficiencies of the model for these regimes may be addressed by use of an extended model.

## Results

### Spectral composition of downwelling illumination during daylight, twilight and night

**Figure 1:**
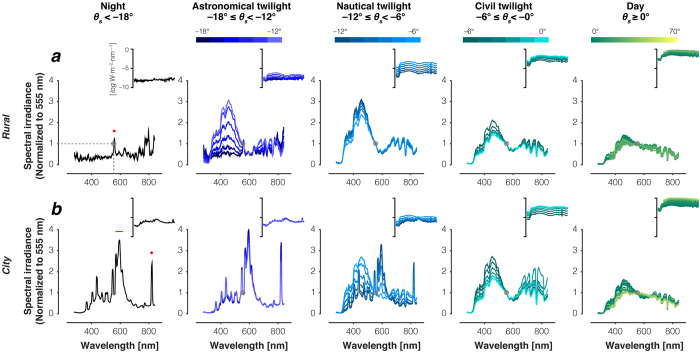
Relative and absolute downwelling illumination at night, twilight and day. (**a**) Measurements in the *Rural* location. Red dot in night time spectrum panel indicates 558 nm. (**b**) Measurements in the *City* location. Red dot in night time spectrum panel indicates 819 nm; red line indicates 570–615 nm. Spectral irradiance measurements were binned by 1° steps of solar elevation and averaged (except for night, where all measurements for θ_s_ < 18° were averaged). The colour bars above the individual plots indicate the solar elevation bins. All measurements were normalised to a value of 1 at 555 nm to emphasise changes in spectral composition (gray dots). Insets show absolute spectral irradiance distribution on a log scale.

**Figure 2:**
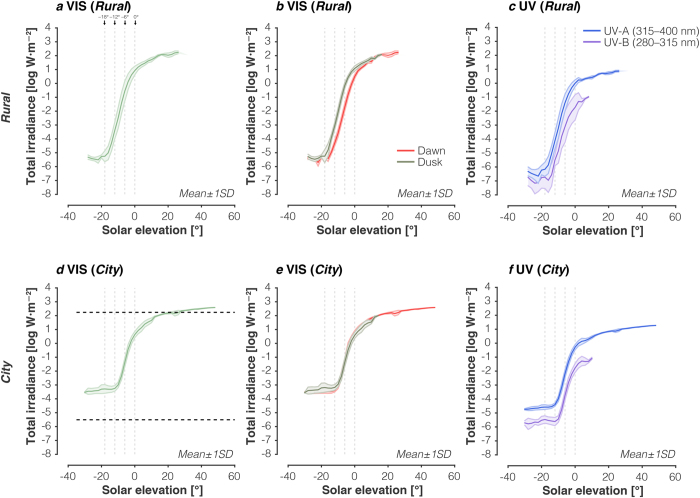
Intensity changes in downwelling illumination as a function of solar elevation. (**a)** Dependence of total visible irradiance (400–800 nm) on solar elevation at the *Rural* location. Vertical lines indicate transition zones between night, astronomical twilight, nautical twilight, civil twilight, and daylight. (**b**) Same as in panel (**a**) but split into morning/dawn and evening/dusk sequences. (**c**) Dependence of total UV-A irradiance (315-400 nm) and UV-B irradiance (280-315 nm) on solar elevation at the *Rural* location. Because of wavelength range differences in the two spectrometers used during the different twilight/daylight regimes, there are no UV-A data for the daylight regime. (**d**) Dependence of total visible irradiance (400–800 nm) on solar elevation at the *City* location. Horizontal lines indicate maxima and minimum mean total irradiance values for the rural location (panel **a**). (**e**) Same as in panel (**d**) but split into morning/dawn and evening/dusk sequences. (**f**) Dependence of total UV-A irradiance (315-400 nm) and UV-B irradiance (280-315 nm) on solar elevation at the *Rural* location. Data were averaged in 2° bins.

Figs 1 and 2 summarise the dataset, averaging spectra by solar elevation, independent of whether the Sun was rising or setting (dawn vs. dusk). Here as in the other main figures and analyses, *Rural* data were restricted to cases where the fraction of the moon illuminated was <0.3, and included all measured spectra independent of lunar phase from the *City* data. At 0.3 fraction of the moon illuminated, the brightness of the moon is approximately 3.29% of the brightness of a full moon^11^. We evaluate the potential effect of moonlight in the measurements below and conclude that it is small.

As solar elevation decreases, the spectra change shape systematically: there is a relative enrichment of short-wavelength light that grows stronger with decreasing solar elevation (Fig. 1a,b) from daylight through to nautical twilight. These changes in spectral composition occur at both the *Rural* and *City* locations; indeed, the average spectral power distributions are quite similar between locations for both daylight and civil twilight. For nautical twilight and astronomical twilight, the data from the two locations diverge. At the *City* location, the dependence on elevation ceases and the spectra do not differ appreciably between astronomical twilight and night (Fig. 1b). In essence the *City* measurements are strongly influenced by artificial illumination starting with nautical twilight, and are dominated by such illumination for astronomical twilight and night. At the *Rural* location, in contrast, the short-wavelength enrichment seen most prominently in nautical twilight (Fig. 1a, middle panel) exists only for the higher elevation spectra in the astronomical twilight regime. These short-wavelength-dominant spectra are then followed by a systematic reduction in short-wavelength power with further decreases in solar elevation. We describe the nature of the night illumination further below.

### Intensity variation as a function of solar elevation

The total irradiance in the visible band (VIS; 400–800 nm) also varies systematically with solar elevation (Fig. 2a,d). Across measurement locations, the relationship between log total irradiance and solar elevation have a similar sigmoidal shape. Total irradiance changes most rapidly with solar elevation during twilight, but is preceded (at dusk) or followed (at dawn) by more gradual changes in overall irradiance. Critically, the lowest total irradiance (*θ*_*s*_ < −18°) in Cherry Springs State Park (Fig. 2a) is about 100 times dimmer than in Philadelphia (Fig. 2d; dashed lines indicate maximum and minimum at the *Rural* location). Next, we considered differences between the dawn and dusk sequences. There are small differences at the *Rural* location (Fig. 2b) but not at the *City* location (Fig. 2e). These differences might arise from different dusk/dawn atmospheric conditions at the *Rural* location that were not present at the urban location, although this is only speculation. We also considered the changes in the UV-A (315-400 nm) and UV-B (280-315 nm) spectral bands (Fig. 2c,f). The dependence of UV intensity on solar elevation is similar to that observed in the visible (VIS). Note that we have incomplete data (Fig. 2c,f; purple line) in the UV-B band because our daylight-level spectrometer did not provide UV-B measurements.

### Night spectra in the *City* and in the *Rural* location

As described above, the night spectra and the astronomical twilight spectra are essentially the same at the *City* location (Fig. 1b) but differ considerably at the *Rural* location (Fig. 1a). The spectra we measured in Philadelphia are dominated by light pollution starting at a solar elevation of about −8° during nautical twilight. Indeed, the average night spectrum is very similar to a previously published spectral measurement of an urban sky in Northern America^1^, suggesting that urban night spectra are generally dominated, in intensity and spectral composition, by low- and high-pressure sodium lamps. Consistent with this, a prominent peak in the twilight and night *City* spectra is seen near the 819 nm emission line associated with sodium lights^15^. The broader peak in the wavelength range 570–615 nm is also consistent with the emission spectra of artificial lights^15^. For the *Rural* location, the average spectrum is irregular with wavelength, but displays a prominent peak near the 558 nm peak of the emission spectrum of atomic oxygen, which is a known feature of airglow under light-pollution-free conditions^16^,^17^,^18^.

### Influence of the moon on the night sky in the *Rural* location and in the *City*

Although our data set does not allow for a full characterisation of the twilight sequence as a function of lunar elevation and lunar phase^11^, we found that the presence of the moon can be detected in the *Rural* spectra for lunar elevations above 0° when we bin by the fraction of the moon illuminated (Fig. S1a). Our ‘full-moon’ spectrum (fraction of the moon illuminated 0.90–1.00) is ~100 times brighter than the night spectrum when there was less moonlight (fraction of the moon illuminated 0.10–0.20 and 0.20–0.30; the latter corresponding to a moon brightness 3.29% of a full-moon^11^; Fig. S1a). At the same time, the *Rural* night spectra do not change appreciably when the fraction of the moon illuminated is reduced from 0.20–0.30 to 0.10–0.20 and the spectra for these low illumination fractions generally have a different spectral shape from the ‘full-moon’ spectrum. These observations suggest that at the low-illumination fractions retained in the *Rural* data (only fraction <0.30 are retained in the main figures and analysis), the moon illumination makes only a small contribution to the measured *Rural* spectra.

The *City* data were collected on dates where the fraction of the moon illuminated was higher than for the *Rural* data (Table 1). At the same time, light pollution increases the lowest intensity of the illumination, so that the relative contribution of moon illumination is smaller. Figure S1b shows the average night spectra from the *City* data for various fractions of moon illuminated. The variation in relative spectra with fraction of moon illuminated is small but not systematic with fraction of moon illumination, suggesting that other factors (for example changes in the amount and quality of artificial light over the course of the night, as might occur because of reduction in household illumination or traffic as the night progresses^19^,^20^) contribute significantly to the variation in night illumination. There is, however, a systematic increase in the overall intensity of the illumination with fraction of moon illuminated (Fig. S1a, inset). Despite this, we decided to retain all of the *City* measurements in the main figures and analysis, because excluding the data for higher fractions illuminated would reduce considerably the number of spectra in the dataset.

The online data supplement (see below under *Open-access data set*) includes information about the moon illumination for each measured spectrum, so that the interested reader may reanalyze the data with other choices of lunar inclusion/exclusion criteria.

### Chromatic variation as a function of solar elevation

**Figure 3:**
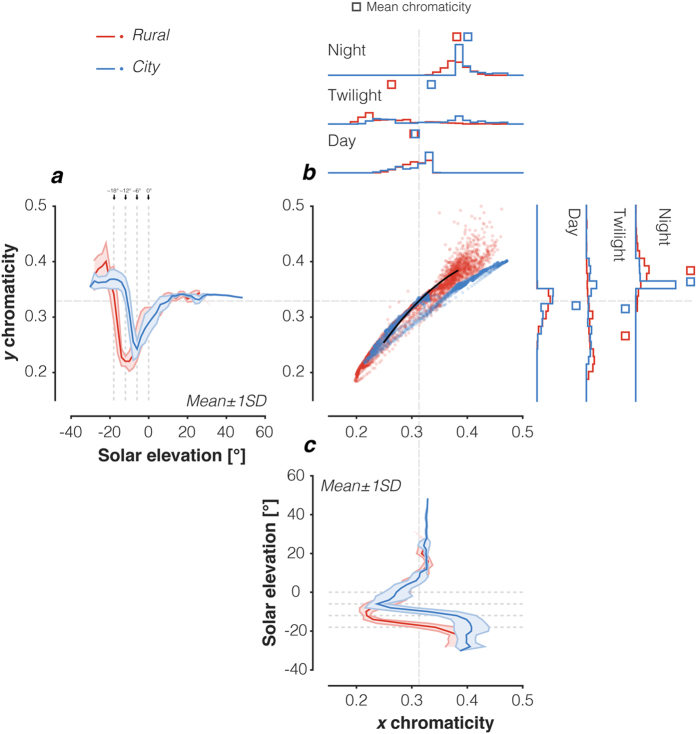
Chromaticity changes in the downwelling illumination as a function of solar elevation. **(a**) Illumination CIE 1931 *y* chromaticity as a function of solar elevation. Vertical lines indicate transition zones between night, astronomical twilight, nautical twilight, civil twilight, and day. The y axis is shared with panel b. Data were averaged in 2° bins. **(b**) Scatter plot of individual chromaticity data for each measured spectrum. The black line indicates the chromaticity along the daylight locus produced by calculating the chromaticity of CIE daylight spectral power distributions with correlated colour temperatures between 4000 K and 25,000 K. Grey cross-hair line indicates the chromaticity of CIE standard illuminant D65 (6500 K). Histograms at the top show the distributions of CIE *x* chromaticity for the daylight, twilight and night regimes, with the x axis shared with panel b. Histograms to the right show the distributions of CIE *y* chromaticity for the daylight, twilight and night regimes, with the y axis shared with panel b. Squares indicate the mean *x* and *y* chromaticities, respectively, within the respective regime. (**c**) Illumination CIE 1931 *x* chromaticity as a function of solar elevation. Horizontal lines indicate transition zones between night, astronomical twilight, nautical twilight, civil twilight, and daylight. The x axis shared with panel **b.** Data were averaged in 2° bins.

**Figure 4:**
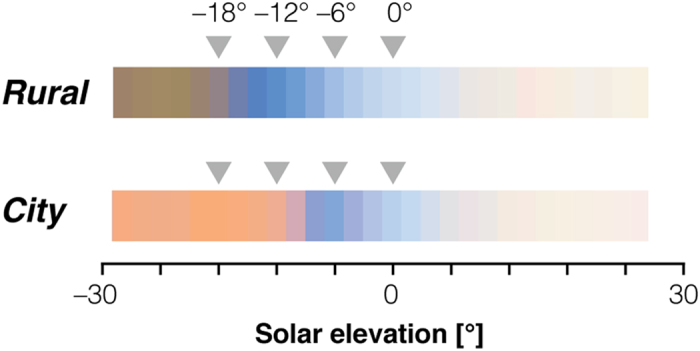
Colour rendering. sRGB pseudo-colour rendering of illuminant spectra. Spectral data were averaged in 2° bins, then normalised by their L^2^-norm. The log (base 10) of the normalizing values were scaled to be within 0.3 and 1, and the L^2^-normalised spectra multiplied by the resulting value. CIE 1931 XYZ tristimulus values were computed from the scaled spectra and transformed to sRGB^32^ values for display.

To further examine variation in illumination with solar elevation, we computed the CIE 1931 *xy* chromaticity coordinates of each measured spectrum (Fig. 3b; daylight locus plotted as black line, coordinates of CIE D65 (daylight with a correlated colour temperature of 6500 K) as dotted grey ‘crosshair’). For both locations, the measurements lie on or near the daylight locus (Fig. 3b), albeit with some spread. Differences between the two locations are most notable during twilight and at night (Fig. 3a,c; also histograms in 3b), while the daylight chromaticities are quite similar. The average twilight chromaticity is shifted towards higher, more reddish values in the *City* location for both *x* and *y* chromaticity. At night, the differences across locations are smaller than during twilight and the *y* chromaticity at the *Rural* location exceeds that in the *City* location, while the *x* chromaticity remains higher, more reddish in the *City* location. We provide colour renderings of the illumination in Fig. 4.

Examining the data in more detail, we note that both *x* and *y* chromaticity ‘dip’ sharply during twilight, with chromaticity shifting towards blue and returning to a more reddish value as solar elevation decreases. The ‘dip’ has a minimum at different solar elevations depending on the measurement location. For the *Rural* location, the illumination is bluest during nautical twilight with a minimum at around −12° and returning towards reddish at lower solar elevations. For the *City* measurements, the ‘dip’ minimum corresponds to higher solar elevations (around −6°).

### CIE daylight model fails to characterise downwelling illumination during twilight

**Figure 5:**
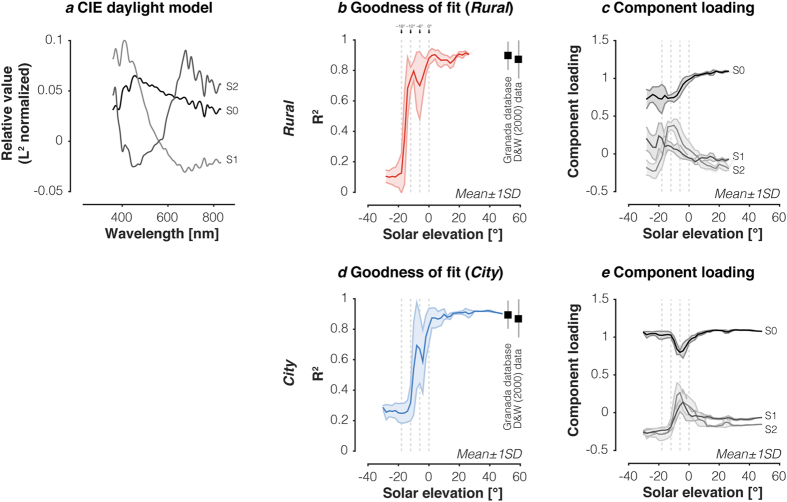
Fits of CIE daylight model to the spectral irradiance measurements. (**a**) CIE daylight basis functions S0 (mean), S1 and S2 between 360 nm and 830 nm. Basis functions were normalised by their vector norm. (**b**) Goodness-of-fit of CIE daylight model as a function of solar elevation in the *Rural* location. The points labelled “Granada database” and “D&W (2000) data” correspond to the mean fit ± 1SD of the daylight model to the daylight spectra from the Granada and DiCarlo & Wandell^3^ datasets. (**c)** Component loadings on S0, S1 and S2 as a function of solar elevation in the *Rural* location (mean ± 1SD). (**d**) Goodness-of-fit of CIE daylight model as a function of solar elevation in the *City* location. The points labelled “Granada database” and “D&W (2000) data” correspond to the mean fit ± 1SD of the daylight model to the daylight spectra from the Granada and DiCarlo & Wandell^3^ datasets. (**e**) Component loadings on S0, S1 and S2 as a function of solar elevation in the *City* location. In panels (**b–e**) vertical lines indicate transition zones between night, astronomical twilight, nautical twilight, civil twilight, and day. Goodness-of-fit values and component loadings were averaged in 2° bins of solar elevation. The CIE model was fit in the 360–830 nm range to the spectra in this dataset, and in the 380–780 nm range for Granada and DiCarlo & Wandell^3^ datasets. R^2^ were calculated in these ranges, respectively.

We examined whether the 3-component CIE daylight model provides an accurate description of our data set at all solar elevations. The CIE daylight model consists of a mean vector S0, and two additional ‘characteristic vectors’ S1 and S2 (Fig. 5a). We fit the three CIE basis functions to each of our spectra and quantified the goodness-of-fit using the coefficient of determination R^2^. As a benchmark, we subjected two large independent data sets to the same analysis: the Granada daylight spectral database consisting of 2,600 daylight spectra collected in Granada, Spain by Hernández-Andrés, *et al.*^21^, and a dataset of 10,756 daylight spectra collected by DiCarlo & Wandell^3^ in Stanford, CA. On average, the CIE daylight model explains 89.6% + 8.8% (mean ± 1 SD) of the variance in the Granada data set and 87.2% ± 12.1% (mean ± 1SD) of the variance in the DiCarlo & Wandell dataset (Fig. 5b,d). We find similar performance of the CIE daylight model during the daylight regime across both of our measurement locations, with an average variance explained of 86.7 ± 7.1% (mean ± 1SD). For twilight and night at both of our measurement locations, however, the CIE model fits degrade rapidly with decreasing solar elevation (Fig. 5b,d). For night spectra, the CIE model explains (on average) less than 10% of the variance for the *Rural* measurements, and less than 20% of the variance for the *City* measurements. In the daylight regime, the component loadings of the basis functions are relatively constant with tight error bounds (Fig. 5c,e). For the twilight and night spectra, component loadings vary more across solar elevation and have wider variation for any given solar elevation.

### Expanding the CIE daylight model to twilight and night regimes

**Figure 6:**
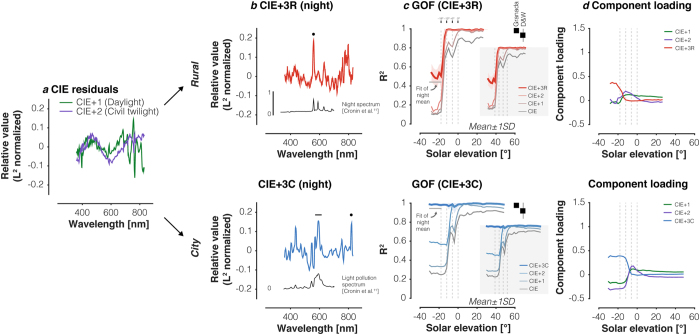
Extending the CIE daylight model to twilight and night illumination. (**a**) Mean residuals of CIE fit for daylight and civil twilight in both *Rural* and *City* location. Residuals were normalised by their vector length. (**b**) Mean residuals after fitting the CIE Model along with the daylight and civil twilight residuals (CIE + 2 model) to the astronomical twilight data from the *rural* location (upper panel) and the *City* location (lower panel). Residuals were normalised by their vector length. Inset in the upper panel shows the normalised night spectrum measured in Zabriskie Point, CA by Cronin, *et al.*^1^. Inset in the lower panel shows the normalised night spectrum measured in Boston, MA by Cronin, *et al.*^1^. Note the correspondence of relative spectral peaks between the residuals and the night spectra measured by Cronin, *et al.*^1^. In the upper panel, black dot indicates 558 nm. In the lower panel, black dot indicates 819 nm; black line indicates 570–615 nm. (**c**) Goodness-of-fit of the full CIE + 3R model in the *Rural* location (upper panel) and the CIE + 3C model the *City* location (lower panel). Vertical lines indicate transition zones between night, astronomical twilight, nautical twilight, civil twilight, and day. Goodness-of-fit values were averaged in 5° bins of solar elevation. Insets in upper and lower panels show goodness-of-fit for the CIE model (same as Fig. 5b,d), and CIE + 1, CIE + 2 and final CIE + 3R and CIE + 3C models. (**d)** Component loadings of the additional three basis functions for the *Rural* and the *City* location. Vert ical lines indicate transition zones between night, astronomical twilight, nautical twilight, civil twilight, and day. Component loadings and goodness-of-fit values were averaged in 2° bins of solar elevation. The CIE, CIE + 1, CIE + 2 and final CIE + 3R and CIE + 3C models were fit in the 360–830 nm range to the spectra in this dataset, and in the 380–780 nm range for Granada and DiCarlo & Wandell^3^ datasets. R^2^ were calculated in these ranges, respectively.

As seen above, the CIE daylight model fails to characterise key features of the illumination at low and negative solar elevations. This is not too surprising, as the CIE model was constructed to capture variation in daylight spectral power distributions. We asked if we could extend the CIE data set to account for twilight and night spectra. We first fit the CIE model to our data and then determined additional basis functions from the residuals (see *Methods*). Note that each of the basis functions corresponds to the average residual in an iterative fitting process that progressively accounts for the daylight, civil twilight, and astronomical twilight spectra. Two of the additional basis functions (CIE + 1 and CIE + 2) were common to both the *Rural* and *City* data sets (Fig. 6a) in the daylight and civil twilight regimes. We determined a third basis function that was derived separately for each location (CIE + 3R*[ural]* and CIE + 3C*[ity]*) from the mean residuals after fitting the extended (CIE + 2) model to the spectra from solar elevations between −18° and −12° (Fig. 6b). The CIE + 3R basis function from the *Rural* location is notable for a prominent peak near 558 nm (Fig. 6b, upper panel), which we previously observed in the mean *Rural* night spectrum and attributed to an oxygen emission line (Fig. 1a). This spectral component is also apparent in a comparable, light-pollution-free night spectrum recorded by Cronin, *et al.*^1^ (Fig. 6b, upper panel inset). The CIE + 3C basis function (Fig. 6b, lower panel) from the city location is notable for spectral peaks from anthropogenic light sources, as was previously noted for the *City* night spectrum (Fig. 1b). This spectral component shares some of the spectral features of a measurement of the city night sky by Cronin, *et al.*^1^ (Fig. 6b, lower panel inset).

With the extended linear models, we can reconstruct key features of the data at both measurement locations with good accuracy. In the *Rural* location (Fig. 6c, upper panel), the CIE + 3R model performs well up to the onset of astronomical twilight. The improved goodness-of-fit of the extended model relative to the 3-component CIE model is apparent for solar elevations between −12 and 0 degrees. The model does not perform well in the nighttime regime, which may be due to the noisiness present in the measurements at these dim light levels. To estimate the noisiness in terms of R^2^, we fit the *Rural* nighttime spectra with the mean nighttime spectrum and computed the R^2^ values to the individual measurements for this simple prediction (Fig. S2). To the extent that the night spectrum at the *Rural* location is in fact unchanging, which we take as a reasonable first-order assumption, this R^2^ value quantifies the measurement variability. It is similar to the R^2^ value obtained with the CIE + 3R model (Fig. 6c, upper panel, horizontal gray lines), which would be expected if the measurement noise is largely orthogonal to the spectral variability captured by the model. We have not pursued this analysis in detail.

In contrast, the CIE + 3C model provides good fits to the *City* spectra at all solar elevations (Fig. 6c, lower panel). Measurement variability was less at the *City* location than at the *Rural* location due the fact that the *City* spectra are more intense than the *Rural* night spectra. Corresponding to this, the R^2^ value obtained when the *City* night spectra are fit with their own mean is high (Fig. 6c, horizontal gray line; Fig. S2). Component loadings on the models behave as one would predict. The components corresponding to the astronomical twilight residuals (Fig. 6d) increase their loading with decreasing solar elevation.

We compared the performance of the CIE + 3R and CIE + 3C models in fitting the Granada daylight data set and the DiCarlo & Wandell daylight data set (Fig. 6c,f) and find that they explain 97.8 ± 1.6% and 97.9 ± 1.9% of the variance (mean ± 1SD), respectively, for the Granada dataset, and 93.1 ± 6.3% and 92 ± 7.9% of the variance (mean ± 1SD) for the DiCarlo & Wandell data set (Fig. 6c,f).

### Model comparisons for all regimes

The Granada dataset has lead to the development of a different set of 6, linear basis functions, which we term here the ‘Granada’ model^21^. We fit the Granada model to our data set and find that it captures daylight, civil twilight, and nautical twilight well but is unable to fit astronomical twilight and night spectra at the *Rural* (Fig. S3a) and *City* locations (Fig. S3b). The CIE + 3R has a marginal advantage in the night regime (Fig. S3a), and the CIE + 3C has a substantial advantage (explaining ~25% more of the variance) at night for the *City* location (Fig. S3b). We note that this is not a surprise, given that the Granada data set (from which the Granada model is derived) is limited to daylight and civil twilight measurements (lowest solar elevation measured in their data set is approximately −4°).

### Daylight-only models

While the goal of the CIE + 3R and CIE + 3C models is to capture daylight, twilight, and night regimes of illumination, we also considered the question of which model would perform best in the daylight regime alone. To this end, we fitted the Granada dataset, the DiCarlo & Wandell data set, and all daylight spectra (solar elevation >0°) with the CIE daylight model, the Granada daylight model, and our CIE + 3R and CIE + 3C models (Fig. S4). On average, the original three-component CIE model performs worst on all data sets (Fig. S4). The Granada model has a marginal advantage in fitting the Granada dataset and a larger advantage in fitting the DiCarlo & Wandell data set. The CIE + 3R and CIE + 3C models fit our daylight spectra well, as expected given that they were generated from this data set, but the difference between these and the fit of the Granada model is very small. Thus, for 6 basis functions, the Granda model provides the best fits for daylight, while our extended models provide the best fits for data across all solar elevations.

## Discussion

In this study, we collected spectroradiometric measurements of downwelling illumination as a function of solar elevation (time of day) in a rural location (Cherry Springs State Park, PA) and in a city location with light pollution (Philadelphia, PA). The spectral and intensity changes of downwelling outdoor illumination largely agree in these two measurement sites up to nautical twilight (−12° < *θ*_*s*_ < −6°). At this point, the dominant illumination source in the city is artificial anthropogenic light. This light pollution truncates the twilight period during which outdoor illumination is enriched in short-wavelength, blue light.

We found that the CIE daylight model did not accurately capture changes in the spectral composition of the illumination at twilight, and to a lesser extent, missed daylight components as well. We therefore created expanded illumination models that added basis functions to the CIE model. Two of these added components were shared between the *Rural* and *City* models. A third component, which captures the lowest-light level spectra, differed across the two models. In an analysis of spectra collected in Granada, Spain, Hernández-Andrés, *et al.*^22^ found that even though three basis functions (such as those proposed by the CIE) were sufficient for colourimetric reconstruction (i.e. in terms of chromaticity coordinates), they suggested that six basis functions are needed for accurate spectral reconstruction of daylight. We also find that we need 6 basis functions here for good spectral reconstruction, but that the 6 basis functions appropriate when twilight and nightime spectra are of interest in addition to daylight are different from those optimised for daylight (Figs S4 and S5). In addition, different basis functions are required for *Rural* and *City* locations (Fig. 6).

Our urban measurements were made on a five-story rooftop that was taller than all other buildings in its immediate vicinity, a location chosen in order to capture a spectrum of light both transmitted through and reflected from the atmosphere over the city and spatially averaged over relatively long (~1 km) distances. This rooftop location therefore provided us a measurement of the average background of urban skylight illumination at night. Given the nature of artificial lighting, the details of street-level measurements at night are hyper-local, varying over length scales of about one-meter, with signals dominated by immediate spatial and temporal proximity to individual street lamps, windows, headlights, etc, making street-level measurements uninformative for our purposes. In addition, we studied only two locations, one rural and one urban, and how our measurements and models generalise to other rural and urban locations is an open question. Examination of nighttime satellite images^23^ suggests that the amount of artificial light in Philadelphia is similar to that in other major cities around the world; determining whether the spectral composition is also similar would require additional data-taking in other cities. However, given that our nighttime measurements share broad features with the spectra reported by Cronin, *et al.*^1^ (Fig. 6) and that our daylight measurements are consistent with extant daylight characterisations provide reason for optimism about the generalisability of our measurements.

This study was not designed to provide systematic measurements of the contribution moonlight as a function of lunar elevation and fraction illuminated, and here we focussed on conditions where the effect of moonlight was small. This is a limitation of our study. In a set of spectral twilight measurements across the lunar cycle, Palmer and Johnsen^11^ found that moonlight can be detected for solar elevations of −8° or less, corresponding to the period of nautical twilight. They note that in the spectra they measured, the effect of moonlight was independent of other light sources, suggesting that the effect of moonlight could be modelled as an additional linear component parameterised by lunar phase.

Ambient illumination acts as a *zeitgeber* (‘time giver’) for photoentrainment of the circadian rhythms of virtually all organisms, affecting many behavioural and physiological processes^24^. Indeed, both intensity and spectral changes appear to be important determinants for the alignment of circadian rhythms to the solar day^10^,^25^. Our data shows that in the *City* location, both changes in intensity and colour of the illumination over the course of the day are affected by light pollution from anthropogenic light sources. Further research could address the effect upon circadian rhythm of alteration of spectral dynamics in the solar day due to light pollution.

## Methods

### Procedure

We measured the *downwelling vector irradiance*, which is defined as the light collected from the entire hemisphere of a measurement surface pointed up. Light arriving at the detector in the direction of the surface is weighted most; the light is integrated according to the cosine of the angle of the incident light^1^,^26^.

Measurements of terrestrial downwelling irradiance spectra under conditions of minimal light pollution were taken in Northern Pennsylvania (Cherry Springs State Park, PA) at different points in the lunar cycle in the months of June and July 2014. Measurements under the new moon were taken between the night of June 30 and the morning of July 4. Full moon measurements were taken on July 11 and 12. Finally, measurements at 60% and 49% fraction of the moon illuminated were taken between July 18 and 20. In the same month, we also collected spectra under conditions of urban light pollution from the roof of a four-story building near the centre of Philadelphia, PA. A summary of the dates and ranges of solar elevation, lunar elevation and fraction of the moon illuminated is given in Table 1.

Spectroradiometric measurements of the downwelling illumination spectrum were taken every 60 seconds. To capture the dynamic range of the illumination, the integration time of the spectroradiometers was adjusted such that the maximum power across all wavelengths did not exceed more than ~85% of the instrument’s maximum allowable intensity reading so as to avoid saturation of the spectral measurements under rapidly changing illumination conditions. These were changed manually by the experimenter. The integration times used were different in the two spectrometers due to their different sensitivities, with up to 60 s for the high-sensitivity spectrometer at night.

### Locations

Measurements of the sky spectral power distribution were performed at *Cherry Springs State Park*, Potter County, Pennsylvania in the United States (41.6646° N, −77.8125° W; elevation 710 m, NED Point Query Service, USGS National Elevation Dataset), a certified ‘IDA International Dark Sky Park’ (*Rural*). The measurement location was on an elevated site within the largely undeveloped Susquehannock State Forest, and was therefore free from direct and indirect anthropogenic light sources. Special care was taken during the measurement periods to mask any stray light from the laptop computer that controlled the spectrometers, using black cloth. A permit to establish a research camp was obtained from the Department of Conservation and Natural Resources, Commonwealth of Pennsylvania.

We also measured spectral power distributions of downwelling irradiance in an urban setting in *Philadelphia, PA* (*City*), from the five-story roof of David Rittenhouse Laboratories, Department of Physics and Astronomy, University of Pennsylvania (39.952237° N, −75.188734° W; elevation 12 m). Because of the wide availability of electric light sources used in urban environments, the measurements taken at this site were measurements of a mixture of natural illumination, light from artificial sources such as street lamps, and reflections of these sources from the built environment. The roof where we conducted measurements was taller than the other roofs in its immediate vicinity, providing our detector a near-full hemispherical view of the sky, and the roof was not directly lit by its own artificial light sources. For these reasons, this measurement location was adequate for obtaining measurements of downwelling irradiance resulting from both natural and anthropogenic sources that were spatially averaged over large distances in the city.

Solar elevation *θ*_*s*_, lunar elevation and lunar phase were extracted for the given topographic coordinates by generating ephemeris tables obtained using the *Multiyear Interactive Computer Alamanac* software^5^ (MICA Version 2.2.2). Ephemeris tables were interpolated linearly to find solar elevation, lunar elevation and lunar phase for each measurement time and location. The reported true elevations thus do not represent apparent elevations that take local atmospheric conditions and refraction into account. Methods for converting elevations from ephemeris tables to approximate apparent elevations are available^27^.

### Measurement devices

All measurements were taken with two customised USB spectrometers (USB2000+, OceanOptics, Inc.; Dunedin, FL), henceforth referred to as the ‘A’ and ‘B’ spectrometers, coupled to a custom-built probe of downwelling irradiance^28^,^29^. The ‘A’ spectrometer was manufacturer-optimised for high sensitivity measurements (Sony ILX511B linear silicon CCD array), reporting between 180 and 875 nm. This was used for twilight and night measurements. The ‘B’ spectrometer was less sensitive with a wavelength range of 340–1025 nm. This was used for daylight measurements. We used a combination of the two spectrometers to span the factor of 10^8^ in diurnal irradiance between midday and night.

To deal with the large number of possible scene orientations in human vision while still detecting a measurable amount of light in low-light twilight and nighttime conditions, we estimated the illuminant for human vision under a given sky as the downwelling irradiance, or light energy impinging on an upward-facing plane, coming from the sky. To construct a probe for downwelling irradiance, we followed the technique described by Sweeney and colleagues and Johnsen and colleagues^28^,^29^. A single-mode, 3-m fibre optic patch cable (1000 μm diameter, 5.8 mm nominal OD, numerical aperture 0.22 ± 0.02, acceptance angle θ_max_ 12.7°, full angle 25.4°; OceanOptics Inc., Dunedin, FL) was connected to the spectrometer and fed through a tube in the downwelling irradiance probe. To construct the probe, the measuring end of the fibre optic cable was pointed at a 45° angle towards a 10.16 cm (4″) diameter plexiglass disk painted with Avian-B White Reflectance Coating (Avian Technologies; New London, NH), a water-based barium sulfate (BaSO_4_) coating. Ultimately, this 45° angle is arbitrary, and all that is required for physically equivalent measurements is that the cable “views” an unoccluded patch of the scattering disk in the same orientation for each measurement. Practically speaking, the choice of 45° splits the difference between the cable potentially viewing its own shadow on the disk when pointed at an angle near 0° from normal, and the cable potentially missing light reflected from the disk with tiny shifts in the mounting hardware when pointed at a glancing angle closer to 90° from normal to the disk. An O-ring fixed around the cable held the distance in which the cable was inserted into the tube and thus its distance to the reflectance disk constant. The distance between the tip of the cable and the center of the reflectance disk was 7.74 cm. We verified that the fibre optic cable could only detect light reflected by the disk by shining a tungsten halogen light (LS-1; OceanOptics, Inc., Dunedin, FL) through the cable in a dark room. When connected to a lamp and not a spectrometer, only the reflectance disk and no other parts of the measurement assembly were illuminated. This indicated that the alignment and positioning of the fibre optic probe were in good order.

A Lenovo Thinkpad X240 laptop computer running Linux distribution Xubuntu 14.04 was used to control the spectrometers and record the measurements. The laptop was equipped with hot-swappable 9-cell batteries, allowing uninterrupted power during the measurements periods. The *OmniDriver* API package (OceanOptics, Inc.; Dunedin, FL) and custom software written for MATLAB (Mathworks Inc.; Natick, MA) was used to read and save spectral power distributions from the spectrometers onto the laptop hard drive.

### Spectrometer calibration

#### Thermal noise and dark calibration

Dark measurements (with a metal cap on spectrometer entrance port) were performed while the two spectrometers were placed in a MyTemp Mini Digital Incubator (Benchmark Scientific; Edison, NJ). The initial temperature in the incubator was 24 °C. The incubator temperature was set to 10 °C, while continuous dark measurements were performed. Then, the incubator temperature was set to 60 °C, continuing to take measurements. The board temperature within the spectrometers, as reported by their internal sensors, was measured along with the dark spectra. This allowed us to create a database for dark spectra parameterised by integration time and measured board temperature, which we found to be the salient parameters in the noise in a given dark spectrum (Fig. S5). The dark spectrum in this library most similar to a given measurement condition was used in processing all individual spectra in the dataset.

#### Wavelength calibration

The USB spectrometers come factory-wavelength calibrated, with each pixel on the sensor corresponding to a wavelength. We validated this calibration with independent wavelength measurement using two line sources (AS-361 Mercury [Hg] Spectral Calibration Lamp; AS-364 Argon [Ar] Spectral Calibration Lamp; Spectral Products, Putnam, CT). We corrected the factory calibration by comparing the measurements to a sample of known spectra lines (404.7, 435.8, 546.1, and 579 nm for mercury source; 696.5, 706.7, 727.3, 738.4, and 763.5 nm for Argon source). These corrections were well-approximated by a single additive shift and were small (<1 nm for both spectrometers), indicating good factory wavelength calibration. The shifts necessary for the two spectrometers were −0.79 ± 0.57 nm (‘A’ spectrometer) and −0.98 ± 0.37 nm (‘B’ spectrometer) on average (±1SD), averaged across spectral lines, respectively (Fig. S6).

#### Absolute irradiance calibration

Although the spectrometers come with factory wavelength calibration, they are uncalibrated with respect to the power read at each wavelength. To bring the measurements into absolute radiometric calibration, we needed to relate a measurement of the absolute spectral irradiance arriving on the disk of the measurement assembly to the raw readings over wavelength of each spectrometer. Because the wavelength ranges of the various calibration sources and measurement instruments available to us were each only partially overlapped with the wavelength ranges of the two spectrometers, we used the following (somewhat involved) procedure.

We took measurements with both spectrometers (‘A’ and ‘B’) of three calibration sources (relative spectra shown in Fig. S7a,b):

1. The reflectance of our measurement sample illuminated with a slide projector (Kodak Carousel 4400; Kodak Inc., Rochester, NY). We also measured this source using a PR-670 spectral radiometer (Photo Research Inc., Chatsworth, CA), which provided the spectral radiance of the same sample in absolute units (W**·**m^−2^**·**sr^−1^**·**nm^−1^) in the wavelength range 380 nm to 780 nm. With this measurement, we obtained the absolute irradiance calibration for both spectrometers across the wavelength range 380–780 nm.
2. A tungsten halogen light source (SL1-CAL; StellarNet, Inc., Tampa, FL) with a corresponding NIST-traceable spectral irradiance measurement at its exit port provided by the manufacturer. This provided relative spectral irradiance calibration at long wavelengths.
3. A deuterium light source (SL3-CAL; StellarNet, Inc., Tampa, FL) with a corresponding NIST-traceable spectral irradiance measurement at its exit port provided by the manufacturer. This provided relative spectral irradiance calibration at short wavelengths.

We investigated the measurement signal-to-noise ratio for each of these three sources as a function of wavelength for both spectrometers. We calculated the absolute correlation coefficients between the set of measured values at odd wavelengths and the set of measured values at even wavelengths within a window of ±20 nm around each wavelength (Fig. S7c,d). In other words, we took the samples within that ±20 nm window and found two disjoint sets within that window and correlated them with each other. In theory, high correlations in such a window point to good signal quality and measurement of the smoothly varying light sources. This was indeed the case (Fig. S7c,d).

We combined the three sets of calibration measurements as follows (see Fig. S7). After filtering with an 8^th^-order one-dimensional median filter using MATLAB’s medfilt1 function (Mathworks Inc.; Natick, MA), correcting for wavelength and subtracting the mean dark signal appropriate for that integration time and temperature, both ‘ground truth’ and measured spectra were interpolated to 1-nm wavelength spacing. Relative correction factors as a function of wavelength are then given as the ratio of the known relative spectrum of the source and the measured spectrum. These correction factors were obtained separately for each source. We then spliced the three sets of correction functions together by choosing 400 nm as a transition point to go from the correction factors derived from the measurements UV-rich SL3-CAL lamp to the correction factors derived from the PR-670 measurements of the assembly sample, and 760 nm as the transition point between the correction factors derived from measurements of the IR-rich SL1-CAL lamp (Fig. S7e,f). The correction factors were brought into absolute scale determined by the PR-670 measurements by finding, in a least-squares fashion, the scalar which brought the SL1-CAL and SL3-CAL-derived correction factors in the ±20 nm range around the transition points into accord with the PR-670 derived correction factors (Fig. S7g,h). The correction factors were then spliced together smoothly using a linear weighting ramp across ±20 nm around the transition points, with an equal weighting of the correction factors at the transition wavelengths (Fig. S7i,j).

#### Processing procedure

The USB spectrometers report uncalibrated spectra that are uncorrected for integration time. To convert these spectra into spectral downwelling irradiance measurements, we followed the following sequence of corrections: 1) For a given uncalibrated raw measurement reported from the USB spectrometers, we first found our best estimate of the dark noise, taken from our dark noise database parameterised by integration time. At a given wavelength band, we interpolated the value as a function of board temperature, and did this for all wavelength bands, yielding a dark noise spectrum that we subtracted from the measurement; 2) we divided the result by the integration time, yielding a noise-corrected measurement in units of uncalibrated power per second; 3) we interpolated from the non-uniform, factory-calibrated wavelength sampling to wavelength sampling at 1 nm between 280 nm and 840 nm (‘A’ spectrometer) and 360 and 840 nm (‘B’ spectrometer) and corrected for the factory wavelength calibration as described above; 4) we then multiplied by the wavelength-dependent radiometric correction factors found using the procedure described above, yielding the spectral radiance of the illumination-measurement assembly sample spectrum in W**·**m^−2^**·**sr^−1^**·**nm^−1^; 5) we converted to spectral irradiance, in W**·**m^−2^**·**nm^−1^ by multiplying radiance by π, which is the projected solid angle over a hemisphere^30^.

#### Quality control and filtering

Any spectra with saturated values at any wavelength were discarded from the analysis. Occasionally, the dark noise subtraction resulted in negative values, which we set to zero for further analysis. We discarded any spectra for which this procedure led to spectra that were zero at all wavelengths. We filtered each spectrum using an 8^th^-order one-dimensional median filter using MATLAB’s medfilt1 function (Mathworks Inc.; Natick, MA).

### Daylight datasets and models

We considered two additional and independent data sets and two alternative models in this paper. We obtained a data set of 10,756 daylight spectra collected by DiCarlo and Wandell^3^ in Stanford, CA from the authors (personal communication), and a data set of 2,600 daylight spectra (henceforth called ‘Granada spectra’) collected by Hernández-Andrés, *et al.*^21^ from the authors’ website (http://colorimaginglab.ugr.es/pages/Data; accessed December 14, 2015). We obtained the CIE basis functions from Wyszecki and Stiles^31^ and the six-component model derived from the Granada spectra (henceforth called ‘Granada model’) from the authors’ website (http://colorimaginglab.ugr.es/pages/Data; accessed December 14, 2015). We digitised the reference rural night sky spectrum from Zabriskie Point, CA and the light pollution spectrum from Boston, MA from Cronin, *et al.*^1^ using WebPlotDigitzer (http://arohatgi.info/WebPlotDigitizer/, accessed December 15, 2015).

### Model fitting procedure

We fitted the various models to the spectral data as follows. We first splined the basis functions to have 1-nm spacing and then normalised each basis function by its vector norm (L^2^-norm). We then separately normalised each measured spectrum by its vector norm and found the best-fitting weights of the basis functions using least-squares regression. We calculated the proportion of variance (R^2^) explained in the measured spectrum by the model fit as the squared linear pairwise correlation coefficient across wavelengths. The procedure described above yields the linear model weights required to fit the normalised spectra. It is these normalised weights that are plotted in the graphs of component loadings (e.g., Fig. 6d). In the data supplement, we provide mean normalised weights grouped by solar elevation (Tables S6 and S7) as well as the mean scale factor required to map the normalised spectra for each elevation group back to absolute spectra. Normalisation, fitting and calculation of R^2^ was performed in the wavelength range 360–830 nm for our data set, and in 380–780 nm for the Granada and DiCarlo & Wandell datasets (see Figs 5 and 6, S2, S3 and S4 captions for details).

### CIE + 3R and CIE + 3C extended CIE models

To capture components in our data set not characterised by the CIE daylight model, we used the following iterative approach. After fitting the CIE daylight basis functions, we extracted the mean residuals for all *Rural* and *City* spectra for daylight (*θ*_*s*_ ≥ 0), and took the average of these residuals to produce the first additional basis function, which we refer to as the CIE + 1 basis function; we refer to the model consisting of the CIE daylight model plus the CIE + 1 basis function as the CIE + 1 model. We then fit all *Rural* and *City* spectra for civil twilight (−6° < *θ*_*s*_ < 0°) with the CIE + 1 model, and found a second additional basis function as the average of the residuals from this fit (CIE + 2 basis function). By adding the CIE + 2 basis function to the CIE + 1 model we obtain the CIE + 2 model. Finally, for the two measurement locations, we added a different, sixth basis depending on the location, extracting the residuals from a fit with the CIE + 2 during astronomical twilight (−18° < *θ*_*s*_ < −12°) separately for each location, and using the average of these residuals as additional basis functions (CIE + 3R*[ural]* and CIE + 3C*[ity]* basis functions). This produced two CIE + 3 models (CIE + 3R and CIE + 3C), one for each location. The decision to use separate basis functions for astronomical twilight at the two locations stems from the observation that this is the point in solar elevation at which the spectral composition begins to differ substantially between the two locations (Fig. 1).

We restricted our analyses to data points for which the lunar fraction was <0.3 in the *Rural* location, and included all spectra independent of lunar phase from the *City* data. At 0.3 fraction of the moon illuminated, the brightness of the moon is approximately 3.29% of the brightness of a full moon^11^. In this data set, apart from a comparison with a full-moon twilight sequence, we did not consider the systematic effect of lunar phase on twilight as we do not have sufficient data that spans the entire lunar cycle.

### Open-access data set

The data supplement contains the *Rural* and *City* irradiance spectra with associated wavelength spacing (Supplementary Tables S1,2–S3), the CIE + 3R (Supplementary Table S4) and CIE + 3C (Supplementary Tables S5) models, as well as the mean ± 1SD weights of these model components as a function of solar elevation (Supplementary Tables S6 and S7). The raw and uncalibrated data set is available at https://dx.doi.org/10.6084/m9.figshare.2009070.v1 and a MATLAB code repository to process these data and reproduce all elements from the figures of this paper is available at https://dx.doi.org/10.6084/m9.figshare.3124759.v1 or https://github.com/spitschan/IlluminationSpectraDataset (MIT License).

## Additional Information

**How to cite this article**: Spitschan, M. *et al.* Variation of outdoor illumination as a function of solar elevation and light pollution. *Sci. Rep.* **6**, 26756; doi: 10.1038/srep26756 (2016).

## Supplementary Material

Supplementary Information

Supplementary Table S1

Supplementary Table S2

Supplementary Table S3

Supplementary Table S4

Supplementary Table S5

Supplementary Table S6

Supplementary Table S7

## Figures and Tables

**Table 1:** Overview of spectral measurements and metadata.

Location	Start/end time		Twilight sequence [E = evening, M = morning]	Solar elevation [°] Min./max.	Lunar elevation [°] Min./max.	Fraction of the Moon Illuminated Min./max.	*n*
*Rural*	30-Jun-2014 18:52:55	01-Jul-2014 01:04:39	E	−26.94–16.73	−27.17–38.55	0.12–0.13	371
	01-Jul-2014 01:05:39	01-Jul-2014 08:08:23	M	−26.94–26.45	−41.19–−16.94	0.13–0.16	404
	01-Jul-2014 17:29:18	02-Jul-2014 04:04:24	E	−27.01–32.46	−43.87–53.96	0.18–0.21	451
	02-Jul-2014 19:38:19	03-Jul-2014 01:04:48	E	−27.09–8.52	−19.06–39.73	0.27–0.28	319
	03-Jul-2014 01:05:48	03-Jul-2014 06:07:19	M	−27.09–4.28	−48.64–−19.24	0.28–0.31	299
	12-Jul-2014 19:09:09	12-Jul-2014 23:50:01	E	−25.69–13.34	−15.30–28.01	0.99–0.99	279
	14-Jul-2014 12:35:07	14-Jul-2014 21:51:36	E	−13.53–70.54	−59.59–−1.93	0.90–0.93	249
	18-Jul-2014 14:41:32	19-Jul-2014 01:05:16	E	−29.19–62.29	−41.35–7.16	0.48–0.54	489
	19-Jul-2014 01:06:16	19-Jul-2014 05:58:09	M	−29.19–0.92	7.35–56.83	0.47–0.48	292
	19-Jul-2014 18:08:44	19-Jul-2014 18:48:58	E	16.49–24.07	−36.88–−34.56	0.41–0.42	39
*City*	05-Jul-2014 19:30:28	06-Jul-2014 01:05:35	E	−27.36–9.84	−2.66–41.20	0.55–0.57	299
	06-Jul-2014 01:06:35	06-Jul-2014 09:33:28	M	−27.36–42.29	−60.39–−2.84	0.57–0.61	487
	06-Jul-2014 19:26:43	07-Jul-2014 06:52:57	E	−27.46–11.95	−56.98–37.62	0.65–0.70	566
	10-Jul-2014 19:23:45	10-Jul-2014 23:44:06	E	−25.04–10.82	6.04–30.31	0.97–0.97	255
	11-Jul-2014 19:49:55	11-Jul-2014 20:41:57	E	−2.72–6.10	1.08–9.43	1.00–1.00	53
	16-Jul-2014 19:13:22	17-Jul-2014 01:05:37	E	−28.84–12.26	−40.82–20.41	0.70–0.73	351
	17-Jul-2014 01:06:37	17-Jul-2014 10:16:50	M	−28.84–49.33	17.14–52.33	0.67–0.70	536
	17-Jul-2014 19:02:59	18-Jul-2014 01:05:03	E	−29.02–14.08	−42.89–13.91	0.59–0.62	333
	18-Jul-2014 01:06:03	18-Jul-2014 06:33:32	M	−29.01–7.23	14.09–56.88	0.57–0.59	319
