# Erratum: Variation of outdoor illumination as a function of solar elevation and light pollution

**DOI:** 10.1038/srep46930

**Published:** 2017-12-22

**Authors:** Manuel Spitschan, Geoffrey K. Aguirre, David H. Brainard, Alison M. Sweeney

Scientific Reports 6: Article number: 26756; 10.1038/srep26756 published online: 06 07 2016; updated: 12 22 2017.

Due to a technical error during the publication of the original version of this Article, the Supplementary Tables S2 and S3 were published in incomplete forms. These errors have been corrected in the Supplementary Information that now accompanies the Article.

