# Supplementary material for: Variation of outdoor illumination as a function of solar elevation and light pollution
Source: Sci Rep. 2016 Jun 7;6:26756. doi: 10.1038/srep26756 (PMC4895134; doi:10.1038/srep26756)
Supplement: Supplementary Information [file srep26756-s1.pdf]

Variation of outdoor illumination as a function of solar elevation and light pollution
Supplementary Information

Manuel Spitschan[1], Geoffrey K. Aguirre[2], David H. Brainard[1‡*], Alison M. Sweeney[3‡*]

[1]Department of Psychology, University of Pennsylvania, Philadelphia, Pennsylvania 19104, USA
[2]Department of Neurology, University of Pennsylvania, Philadelphia, Pennsylvania 19104, USA
[3]Department of Physics and Astronomy, University of Pennsylvania, Philadelphia, Pennsylvania 19104, USA
[‡]Joint corresponding authors

*Correspondence should be addressed to David H. Brainard or Alison M. Sweeney:

David H. Brainard
University of Pennsylvania, Department of Psychology
3401 Walnut St., Suite 400A
Philadelphia, PA, 19104
brainard@psych.upenn.edu

Alison M. Sweeney
University of Pennsylvania, Department of Physics & Astronomy
David Rittenhouse Laboratory
2N10, 209 S. 33rd St.
Philadelphia, PA 19104
alisonsw@physics.upenn.edu

Supplementary Figure and Table Legends

Figure S1 | Effect of moon illumination on night spectra. **a,** Mean normalised *Rural* night spectra ($\theta_s < 18°$) for four binned lunar phases (see legend). Inset: Absolute irradiance spectra plotted on logarithmic scale. Format as in Fig. 1. **b,** Mean normalised *City* night spectra ($\theta_s < 18°$), same format as **a**.

Figure S2 | Mean night data fits. **a,** Goodness-of-fit (R^2) of the mean night spectrum (shown in panel **b**) in the *Rural* location as a function of solar elevation. **b**, Normalised mean night spectrum from the *Rural* location. **c,** Goodness-of-fit (R^2) of the mean night spectrum (shown in panel **d**) in the *Rural* location as a function of solar elevation. **d**, Normalised mean night spectrum from the *Rural* location. Data were fit in the 360-830 nm range.

Figure S3 | Granada model fits to *Rural* and *City* data. **a,** Goodness-of-fit (R^2) of the six-component Granada model to the *Rural* data as a function of solar elevation. **b,** Goodness-of-fit (R^2) of the six-component Granada model to the *City* data. Data were fit in the 380-780 nm range.

Figure S4 | CIE, Granada, CIE+3R and CIE+3C model fits to daylight data. **a,** Goodness-of-fit (R^2) of the CIE model, the Granada model, and the two CIE+3R and CIE+3C models to the Granada daylight data set, the DiCarlo & Wandell (2000) dataset and daylight spectra from this dataset. Data were fit in the 380-780 nm range.

Figure S5 | Dark noise properties of the spectrometers. Dark noise spectra are shown as a function of board temperature (columns) and integration time (rows) for the 'A' spectrometer (panel A) and 'B' spectrometer (panel B). Spectra are shown as un-normalised, raw read-outs from the spectrometer.

Figure S6 | Wavelength calibration. Measured locations of spectral line peaks (based on factory wavelength calibration) from two line sources for the 'A' spectrometer (panel a) and the 'B' spectrometer (panel b) plotted against nominal locations of these peaks. Insets indicate, for each of the nominal spectral peaks, the measured peak location determined in the wavelength calibration, and the mean±1SD shift required to bring the measured peak values into register with their nominal counterparts.

Figure S7 | Spectral irradiance calibration. **a, b**, Uncalibrated, dark-subtracted, wavelength-corrected and normalised power measurements of the three calibration sources. **c, d**, Correlation coefficients of odd and even wavelength measurements for across a window of ±20 nm around each wavelength. Colour differentiates between light sources; colour scheme as in panels **a, b**. **e, f**, Weighting function of the correction factors. **g, h**, Correction factors determined for each light source. The correction factors for the SL-1 and SL-2 light source were scaled to match those derived from the PR-670 measurements. **i, j**, Final composite correction factors derived from the individual source correction factors shown in panels **g, h** and the weighting function shown in panels **e, f**.

Table S1 | Table of wavelengths. CSV file containing the wavelength spacing for the spectra in Tables S2 and S3 (280-840 nm with 1 nm spacing).

Table S2 l Data from the *Rural* location. This CSV file contains 3,192 irradiance spectra measured at Cherry Springs State Park, PA and associated metadata. Row 1: Date string in '*dd-mmm-yyyy HH:MM:SS*' format. Row 2: Date number. Row 3: Solar elevation [°]. Row 4: Lunar elevation [°]. Row 5: Fraction of the Moon Illuminated [proportion, 0-1]. Rows 6-566 contain the spectral irradiance as $W \cdot m^{-2} \cdot nm^{-1}$ according to the wavelength vector given in Table S1. NaN (= 'not a number') values indicate out of wavelength range for spectrometer used.

Table S3 l Data from the *City* location. This CSV file contains 3,199 irradiance spectra measured at Philadelphia, PA and associated metadata. Row 1: Date string in '*dd-mmm-yyyy HH:MM:SS*' format. Row 2: Date number. Row 3: Solar elevation [°]. Row 4: Lunar elevation [°]. Row 5: Fraction of the Moon Illuminated [proportion, 0-1]. Rows 6-566 contain the spectral irradiance as $W \cdot m^{-2} \cdot nm^{-1}$ according to the wavelength vector given in Table S1. NaN (= 'not a number') values indicate out of wavelength range for spectrometer used.

Table S4 l Tabulation of CIE+3R (*Rural*) model. CSV file. Column 1: Wavelength vector (360-830 nm with 1 nm spacing). Column 2: CIE S0. Column 3: CIE S1. Column 4: CIE S2. Column 5: CIE+1. Column 6: CIE+2. Column 7: CIE+3R. Each basis function is normalised to have unit L^2 norm.

Table S5 l Tabulation of CIE+3C (*City*) model. CSV file. Column 1: Wavelength vector (360-830 nm with 1 nm spacing). Column 2: CIE S0. Column 3: CIE S1. Column 4: CIE S2. Column 5: CIE+1. Column 6: CIE+2. Column 7: CIE+3C. Each basis function is normalised to have unit L^2 norm.

Table S6 l Best-fitting weights of CIE+3R model to *Rural* spectra as a function of solar elevation. Column 1: Solar elevation [°]. Columns 2 & 3: Mean±1SD scalar to map into absolute irradiance. Columns 4 & 5: Mean±1SD weight of CIE *S0*. Columns 6 & 7: Mean±1SD weight of CIE *S1*. Columns 8 & 9: Mean±1SD weight of CIE *S2*. Columns 10 & 11: Mean±1SD weight of CIE+1. Columns 12 & 13: Mean±1SD weight of CIE+2. Columns 12 & 13: Mean±1SD weight of CIE+3R. Values were binned between –30° and 74° of solar elevation, with 2° spacing. NaN (= 'not a number') values indicate that no data were available at that solar elevation.

Table S7 l Best-fitting weights of CIE+3C model to *City* spectra as a function of solar elevation. Column 1: Solar elevation [°]. Columns 2 & 3: Mean±1SD scalar to map into absolute irradiance. Columns 4 & 5: Mean±1SD weight of CIE *S0*. Columns 6 & 7: Mean±1SD weight of CIE *S1*. Columns 8 & 9: Mean±1SD weight of CIE *S2*. Columns 10 & 11: Mean±1SD weight of CIE+1. Columns 12 & 13: Mean±1SD weight of CIE+2. Columns 12 & 13: Mean±1SD weight of CIE+3C. Values were binned between –30° and 74° of solar elevation, with 2° spacing. NaN (= 'not a number') values indicate that no data were available at that solar elevation.

Figure S1

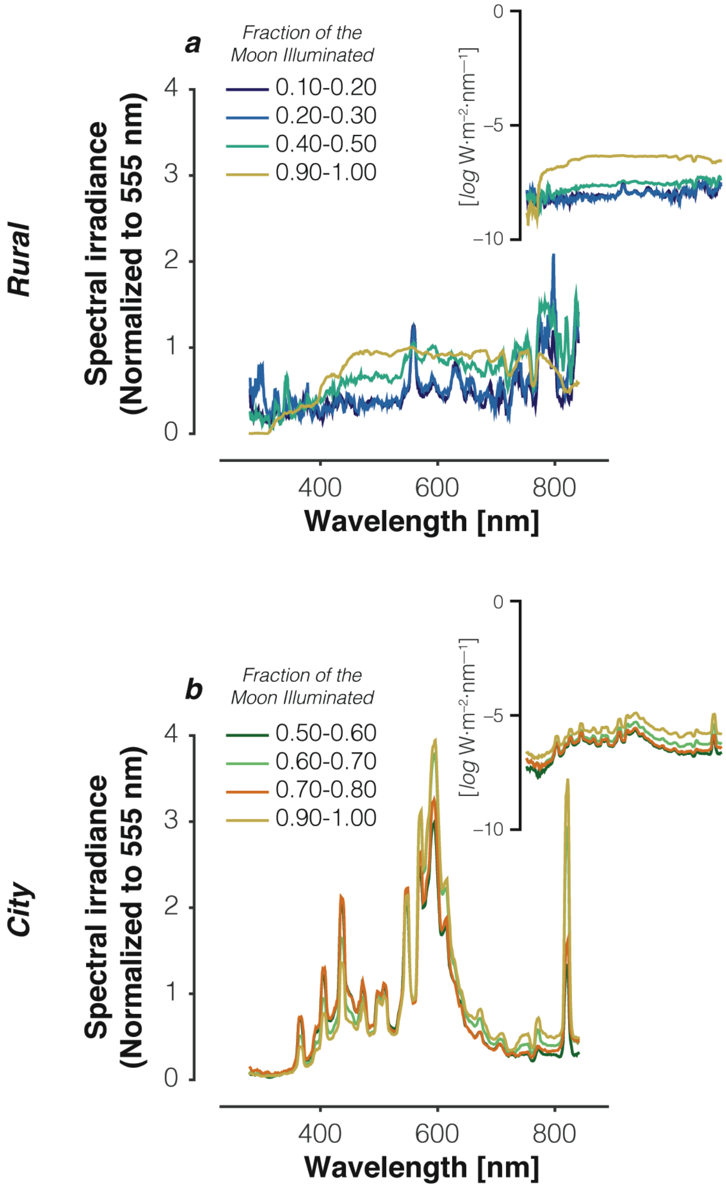
a
Fraction of the Moon Illuminated
0.10-0.20
0.20-0.30
0.40-0.50
0.90-1.00
Rural
Spectral irradiance (Normalized to 555 nm)
Wavelength [nm]
[log W·m⁻²·nm⁻¹]
b
Fraction of the Moon Illuminated
0.50-0.60
0.60-0.70
0.70-0.80
0.90-1.00
City
Spectral irradiance (Normalized to 555 nm)
Wavelength [nm]
[log W·m⁻²·nm⁻¹]

Figure S2

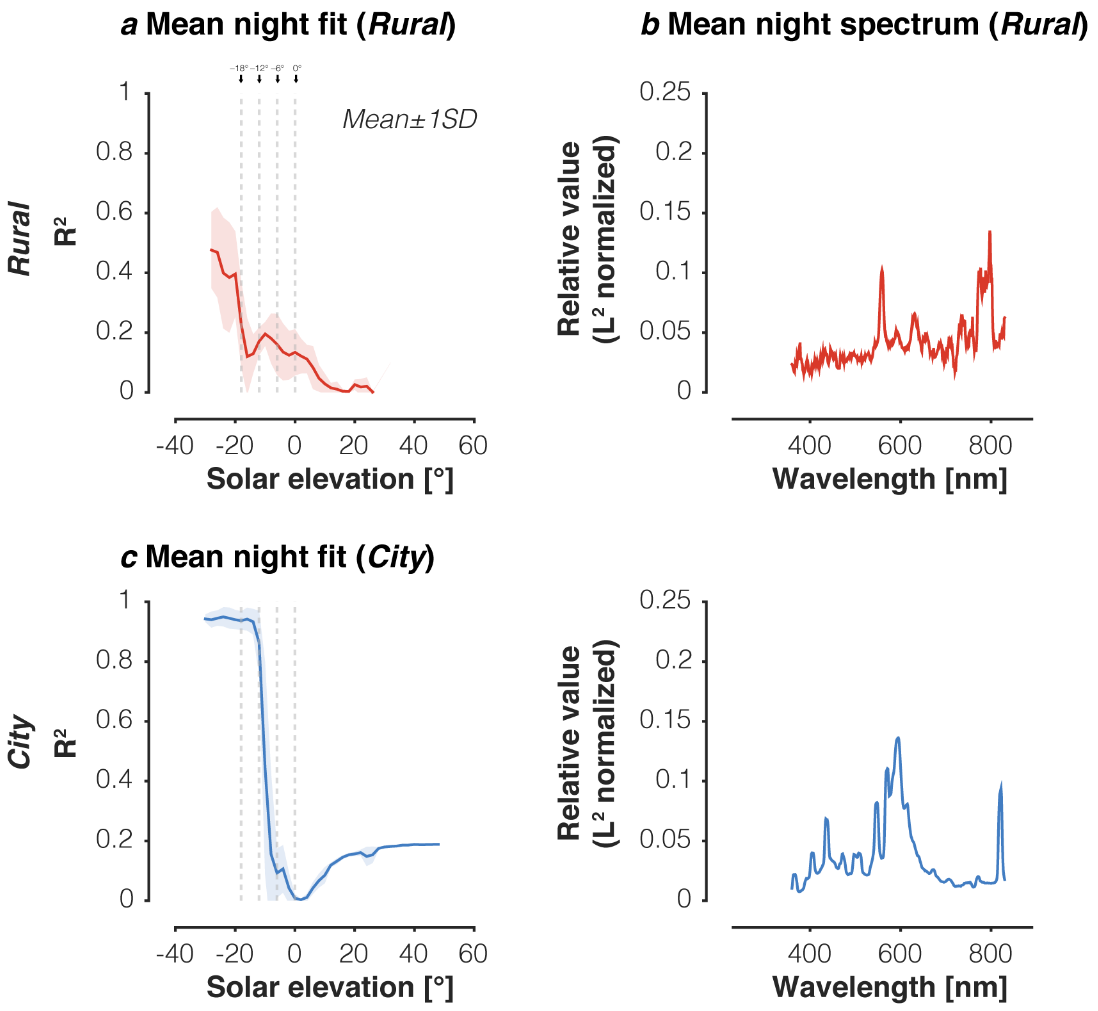

a Mean night fit (Rural)
b Mean night spectrum (Rural)
−18° −12° −6° 0°
Mean±1SD
Rural
R²
1
0.8
0.6
0.4
0.2
0
-40 -20 0 20 40 60
Solar elevation [°]
Relative value
(L² normalized)
0.25
0.2
0.15
0.1
0.05
0
400 600 800
Wavelength [nm]
c Mean night fit (City)
City
R²
1
0.8
0.6
0.4
0.2
0
-40 -20 0 20 40 60
Solar elevation [°]
Relative value
(L² normalized)
0.25
0.2
0.15
0.1
0.05
0
400 600 800
Wavelength [nm]

Figure S3

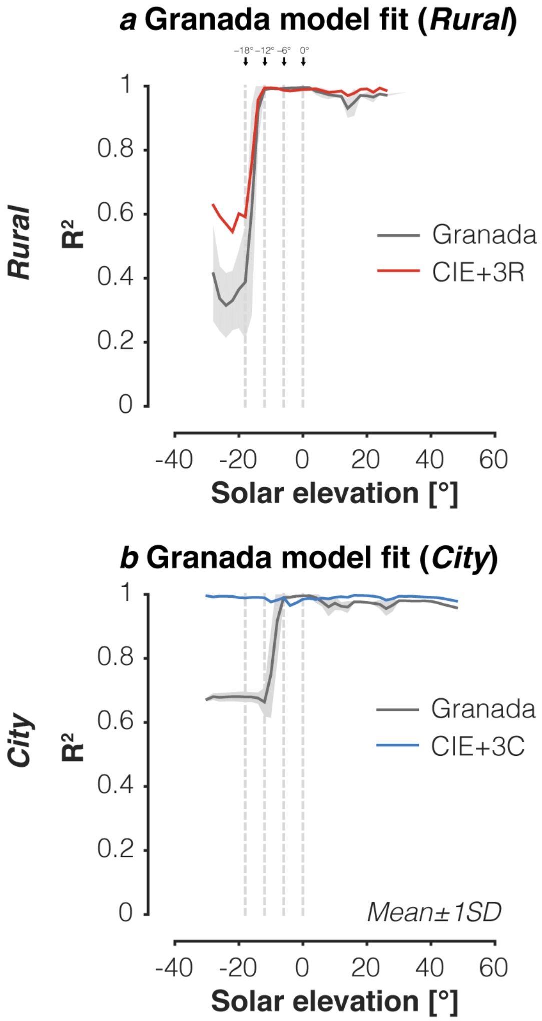
a Granada model fit (Rural)
−18° −12° −6° 0°
Rural
R^2
1
0.8
0.6
0.4
0.2
0
Granada
CIE+3R
-40 -20 0 20 40 60
Solar elevation [°]
b Granada model fit (City)
City
R^2
1
0.8
0.6
0.4
0.2
0
Granada
CIE+3C
Mean±1SD
-40 -20 0 20 40 60
Solar elevation [°]

Figure S4

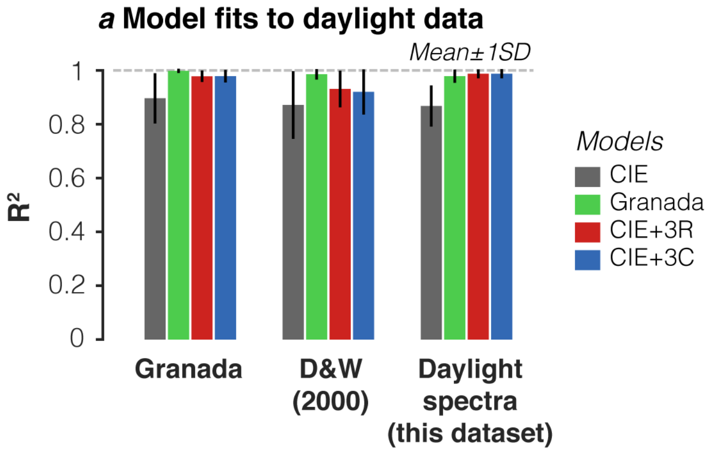

a Model fits to daylight data
Mean±1SD
1
0.8
0.6
0.4
0.2
0
R²
Granada
D&W (2000)
Daylight spectra (this dataset)
Models
CIE
Granada
CIE+3R
CIE+3C

Figure S5

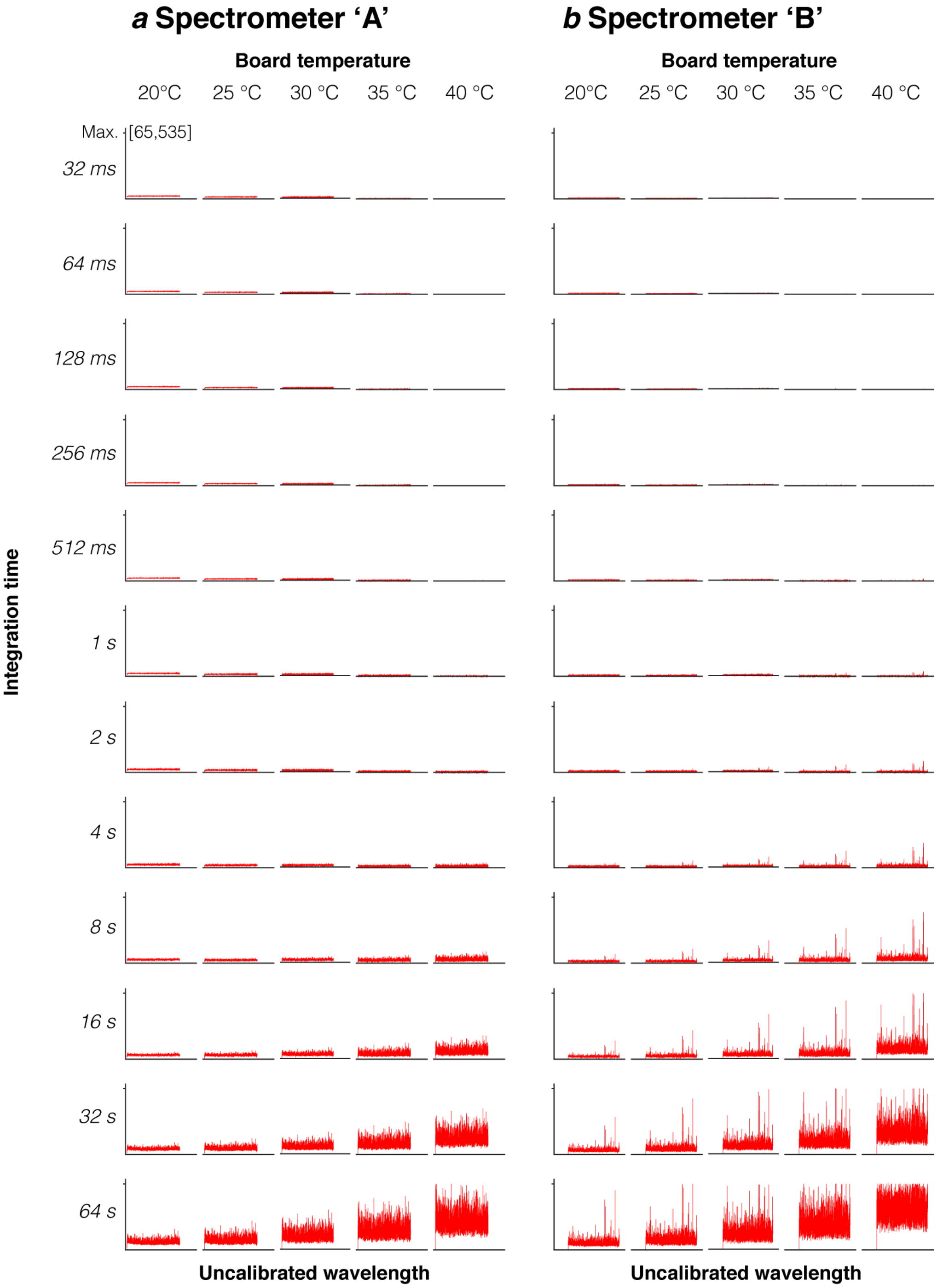

a Spectrometer 'A'
b Spectrometer 'B'
Board temperature
20°C
25 °C
30 °C
35 °C
40 °C
Max.
[65,535]
Integration time
32 ms
64 ms
128 ms
256 ms
512 ms
1 s
2 s
4 s
8 s
16 s
32 s
64 s
Uncalibrated wavelength

Figure S6

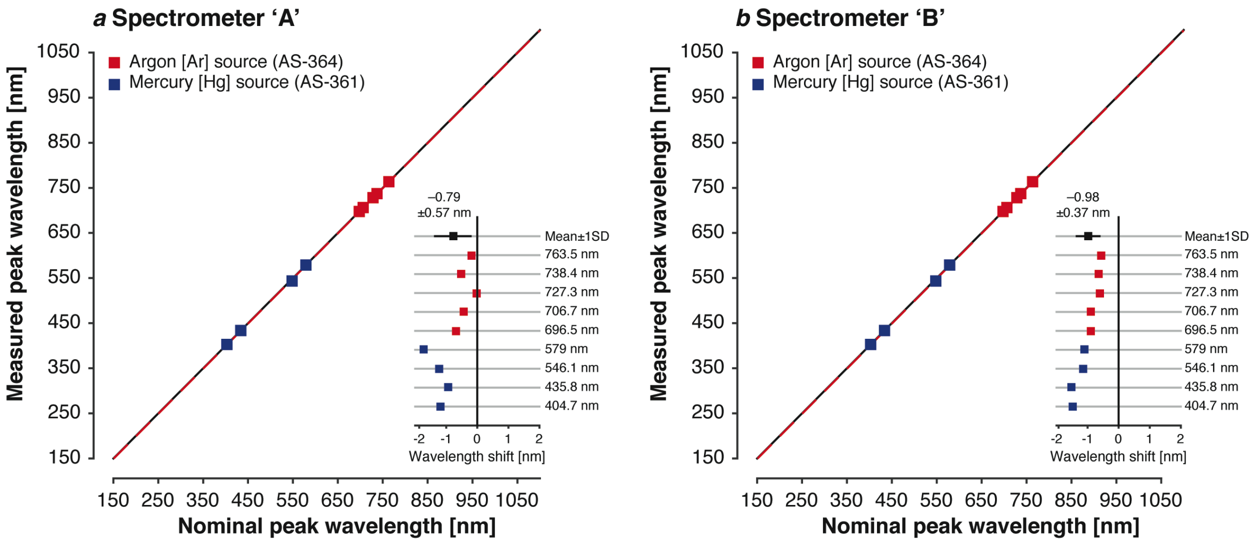
a Spectrometer 'A'
Argon [Ar] source (AS-364)
Mercury [Hg] source (AS-361)
Measured peak wavelength [nm]
Nominal peak wavelength [nm]
150
250
350
450
550
650
750
850
950
1050
−0.79
±0.57 nm
Mean±1SD
763.5 nm
738.4 nm
727.3 nm
706.7 nm
696.5 nm
579 nm
546.1 nm
435.8 nm
404.7 nm
-2
-1
0
1
2
Wavelength shift [nm]
b Spectrometer 'B'
Argon [Ar] source (AS-364)
Mercury [Hg] source (AS-361)
Measured peak wavelength [nm]
Nominal peak wavelength [nm]
−0.98
±0.37 nm
Mean±1SD
763.5 nm
738.4 nm
727.3 nm
706.7 nm
696.5 nm
579 nm
546.1 nm
435.8 nm
404.7 nm
Wavelength shift [nm]

Figure S7

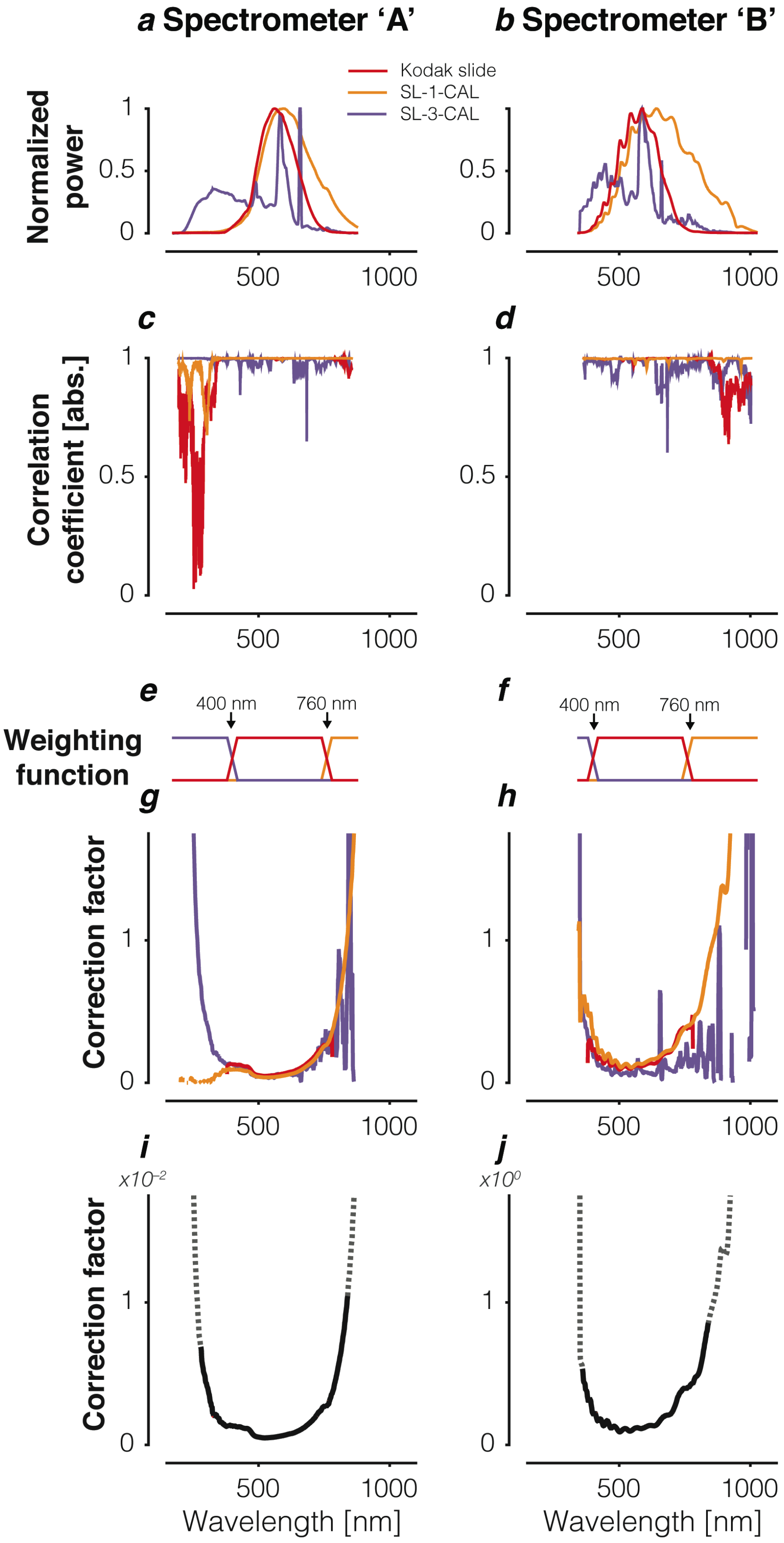
a Spectrometer 'A'
b Spectrometer 'B'
Kodak slide
SL-1-CAL
SL-3-CAL
Normalized power
0
0.5
1
500
1000
c
d
Correlation coefficient [abs.]
e
f
400 nm
760 nm
Weighting function
g
h
Correction factor
i
j
x10⁻²
x10⁰
Wavelength [nm]
